# An extension of the acceptance model of intuitive eating in adolescent girls: a role for social comparison?

**DOI:** 10.1186/2050-2974-2-S1-O40

**Published:** 2014-11-24

**Authors:** Rachel Andrew, Marika Tiggemann, Levina Clark

**Affiliations:** 1 School of Psychology, Flinders University, Adelaide, Australia

## 

Intuitive eating (i.e., eating in response to internal cues) has been shown to be negatively related to disordered eating in women. The acceptance model of intuitive eating proposes that intuitive eating results from increased body appreciation, lowered self-objectification and body acceptance by others. As yet, this model has not been tested in adolescent girls; a group vulnerable to eating pathology. In addition to testing the acceptance model in adolescent girls, this study aimed to examine the role of social comparison. Participants were 400 girls aged 12 to 16 years who completed questionnaire measures of perceived body acceptance by others, social comparison, self-objectification, body appreciation and intuitive eating. In support of the model, perceived body acceptance and body appreciation correlated positively with intuitive eating, while self-objectification and social comparison correlated negatively. Structural Equation Modeling showed the extended acceptance model was an acceptable to good fit to the data. In particular, the effects of perceived body acceptance were mediated by self-objectification and social comparison. The findings replicate the acceptance model of intuitive eating in adolescent girls, but also indicate that social comparison is important in this process. Practically, the findings highlight several areas that may be targeted to foster adaptive eating patterns in girls.

This abstract was presented in the **Prevention & Public Health** stream of the 2014 ANZAED Conference.

